# Psychometric properties of the IDS-SR_30 _for the assessment of depressive symptoms in spanish population

**DOI:** 10.1186/1471-2288-11-131

**Published:** 2011-09-21

**Authors:** Margalida Gili, Juan V Luciano, Natalia Bauzá, Jaume Aguado, María J Serrano, Silvia Armengol, Miquel Roca

**Affiliations:** 1 Institut Universitari d'Investigació en Ciències de la Salut (IUNICS), University of Balearic Islands, Cra. de Valldemossa (km 7,5), Palma de Mallorca (07122), Spain; 2 Red de Actividades Preventivas y Promoción de la Salud en Atención Primaria (RediAPP), Av. Gran Via de les Corts Catalanes, 587 Ático, Barcelona (08007), Spain; 3 Almirall Medical Department, General Mitre 151, Barcelona (08022), Spain; 4 Unitat de Recerca i Desenvolupament, Parc Sanitari Sant Joan de Déu, Doctor Antoni Pujadas 42, Sant Boi de LLobregat, Barcelona (08830), Spain; 5 Institut d'Investigació en Atenció Primària Jordi Gol (IDIAP), Grup de Recerca en l'Atenció Primària i Salut Mental (GRAPISAM), Av. Gran Via de les Corts Catalanes, 587 Ático, Barcelona (08007), Spain

## Abstract

**Background:**

Due to the high prevalence of depression, it is clinically relevant to improve the early identification and assessment of depressive episodes. The main objective of the present study was to examine the psychometric properties of the IDS-SR_30 _(Self-rated Inventory of Depressive Symptomatology) in a large Spanish sample of depressive patients.

**Methods:**

This prospective, naturalistic, multicenter, nationwide epidemiological study conducted in Spain included 1595 adult patients (65.3% females) with a DSM-IV Major Depressive Disorder (MDD. IDS-SR_30 _and the Hamilton Depression Rating Scale (HDRS, 21 items)were administered to the sample. Data was collected during 2 routine visits. The second assessment was carried out after 10 ± 2 weeks after first assessment.

**Results:**

The IDS-SR_30 _showed good internal consistency (α = 0.94) and high item total correlations (≥ 0.50) were found in 70% of the items. The convergent validity was 0.85. Results of the principal component analysis (PCA) and confirmatory factor analyses (CFA) showed that a three factor model (labelled mood/cognition, anxiety/somatic and sleep) is adequate for the current sample.

**Conclusions:**

The Spanish version of the IDS-SR_30 _seems a reliable, valid and useful tool for measuring depression symptomatology in Spanish population.

## Background

Depression is one of the most common diagnosis, with nearly 17% of the adult population in the community meeting criteria for major depressive disorder (MDD) during their lifetime [1] and approximately 7% experiencing MDD during a 12-month period [2]. Among primary care patients depression rates are higher due to the association of chronic disorders [3] Recent epidemiological studies in Spain show prevalences in primary care settings that range from 9 to 29% [4-6].

Due to the high prevalence of this disorder, it is clinically relevant to improve the early identification and assessment of depressive episodes. Thus reliable, valid and brief instruments for the screening of depression are highly required [7].

There are several accepted clinician-rated and patient self-reported measures of depressive symptoms. The most commonly clinician-rated scales used both in the clinical and research context are the 17, 21, 24, 28, and 31-item versions of the Hamilton Rating Scale for Depression (HDRS) [8], and the Montgomery-Asberg Scale (MADRS) [9]. Among the most frequently used self-reports we find the 13 and 21-item version of the Beck Depression Inventory (BDI) [10], the Zung Depression Rating Scale [11], The Carroll Rating Scale (CRS) [12], and the Patient Health Questionnaire-9 (PHQ-9) [13].

New scales for the assessment of depressive symptoms are continuously designed in order to improve the psychometric deficits of the existing instruments. Despite the increasing availability of these new tools, the Hamilton Rating Scale for Depression (HRSD) -first published back in 1960 [8], remains the most used instrument in clinical settings. However, some authors have criticized the incomplete assessment of depressive symptoms as well as the psychometric deficits of this widely used scale [14,15].

The Inventory of Depressive Symptomatology (IDS) [14,16] is an assessment tool that can be used to screen or assess the severity of depression and is widely used in large national and international multicentre studies and clinical trials [17-21]. The time frame for assessing symptom severity is usually a seven-day period prior to the evaluation. The IDS is available in two versions: a clinician-rated (IDS-C) and a self-report (IDS-SR) scale. Both versions require minimal training and are sensitive to changes with medication, psychotherapy, or somatic treatments, making them useful for both research and clinical purposes. These scales have been translated into different languages (German, French, Italian, Chinese...) and a psychometric validation have been published in some of these versions [14,22-26].

The IDS was developed to provide equivalent weightings (0-3) for each symptom item, clearly stated anchors that estimate the frequency and severity of symptoms (including all the DSM-IV criteria required to diagnose a major depressive episode) as well as matched clinician and patient ratings [14,16,27,24]. A 16-item version was later developed and have been examined in adult and young population [28,29].

The self-rated IDS (IDS-SR_30_), the IDS clinician version (IDS-C), and the Quick Inventory of Depressive Symptomatology (QIDS) (both clinician and self-rated) include items that rate the nine symptoms domain used to define a major depressive episode (DSM-IV criteria). The IDS includes additional items to define melancholic and atypical symptom features, as well as commonly symptoms associated to depression (irritability, anxiety).

During the development of the original version, the item-selection of the IDS was aligned with the DSM criteria and other existing depression scales. Furthermore, symptoms of the anxious and melancholic subtypes of depression and other atypical symptoms were included. The selection process was supported by clinical experts and by patients [16]. In its final form, the IDS is composed by 30 items and the total score range from 0 to 84. All items are rated on a scale from 0 (symptom is not present) to 3 (strongest impairment). In the self-rated version (IDS-SR) a cut-off-point of 18 or above indicates the presence of clinical relevant depressive symptomatology [14].

There is published evidence of acceptable psychometric properties for the IDS in the evaluation of depressive outpatients [14,27,24] and inpatients [22]. There is also a substantial correlation between total scores of the IDS-C_30_, the IDS-SR_30 _and the HDRS_17 _. The IDS scores have been useful to differentiate endogenous from non-endogenous depressions [30] and dysthymic disorder from major depressive disorder [16].

The aim of this study is to assess the psychometric properties of the Spanish version of the IDS (self-report) using the Hamilton Depression Rating Scale (HDRS) as a gold standard to detect the presence of a single or recurrent major depressive episode and to determine depressive symptoms severity.

## Methods

### Study design, sampling and recruitment

In the present study we utilized the RESIST data set. The RESIST was a prospective, naturalistic, multicenter, nationwide epidemiological study. The main objective of the RESIST study was to compare residual symptoms between early and late remission in a large sample of depressive psychiatric outpatients in daily routine practice [31]. A regionally stratified sample of 400 psychiatrists was selected to participate in the study. They were proportionally distributed by regions within Spain's 17 regional communities. Each participant was asked to recruit 4 or 5 eligible outpatients. Patients were eligible for the study if they were 18 years or older, they met MDD diagnoses according to DSM-IV criteria and they had 6-8 weeks of antidepressant treatment.

### Participants

A total of 1870 patients were initially recruited. 275 patients were excluded in the final analysis due to different reasons: change of treatment (9.1%), patients without second assessment (3.6%) incomplete or missing data (1.9%). Finally 1595 patients were included in the analysis.

### Procedure

Data collection took place from February to June 2009 after receiving the approval of the Clinical Research Ethics Committee of the Teknon Foundation. Data was collected during 2 routine visits after obtaining written consent from patients. As the RESIST study was originally designed to evaluate the residual symptoms of depression, it was required that patients had been treated with antidepressant at least 6 weeks to have depressive patients in remission at first assessment. Assessments were carried out after 6-8 weeks of antidepressant treatment and at 10 ± 2 weeks after first assessment. Treatment with any antidepressant was allowed by the study protocol. The antidepressant prescribed and the dose was entirely at the discretion of the psychiatrist. Change of treatment resulted in exclusion from the study. Interviews were done by clinical psychiatrists. Self-reports were completed on the same day during the clinical interview.

### Instruments

Case report form at first assessment was fulfilled by the psychiatrist and included the criteria of DSM-IV for major depressive episode that patients should meet. It included also collection of data about sociodemographic characteristics (age, gender, marital status, employment status, educational level, residence environment), and clinical features of the depressive disorder (age of first depressive episode, duration of episode, number of previous episodes, DSM-IV-TR comorbid psychiatric diagnoses and comorbid medical diseases).

The Spanish translation of the Self-rated Inventory of Depressive Symptomatology (IDS- SR_30_) [14] was obtained from the authors and available on the website http://www.ids-qids.org. There was a South American version that we adapted to the current use of the language in Spain.

The 21 item HDRS (Hamilton Depression Rating Scale) [8]: is a 21 question multiple choice questionnaire which rates the severity of symptoms observed in depression such as low mood, agitation, anxiety and weight loss. The clinician must choose the possible responses to each question by interviewing the patient and by observing the patient's symptoms. Each item has between 3-5 possible responses which increase in severity. The first 17 questions contribute to the total score and question 18-21 are recorded to give further information about depression such as if paranoid symptoms are present. The HDRS was used as a gold standard.

### Statistical analyses

The Statistical Package for Social Sciences (SPSS) version 19.0 and the Mplus v5.1 were used to carry-out the statistical analyses.

Factor analyses. We made use of first assessment IDS-SR_30 _scores for a principal component analysis (PCA). Then, a set of confirmatory factor analyses (CFAs) based on the exploratory result and on the scientific literature were performed on a new data set (second assessment scores) to find the best fitting factor structure for the instrument. Following the common assumption that the ratio of subjects per variable (item) is central for factor analysis, in both study periods we were able to satisfy the minimum of five participants-per-item ratio recommended by Kass and Tinsley [32].

Firstly, in order to make our results in the PCA comparable with those recently reported by Wardenaar [33], we performed a Horn's parallel analysis [34] to determine the number of factors to be retained. This is a Monte-Carlo based simulation method, considered more replicable than the traditional extraction techniques (Kaiser's criterion or Scree plot), that compares the unrotated eigenvalues to eigenvalues from a random sample with the same number of cases and variables. PROMAX was used for oblique rotation. To assess the suitability of the data for factor analysis, the Kaiser-Mayer-Olkin's (KMO) Measure of Sampling Adequacy [35] was computed. KMO scores above 0.90 are considered excellent. The Bartlett's test of Sphericity [36] was also applied to examine the extent to which the correlation matrices departed from orthogonality.

Secondly, we tested the fit of the following factor structures: The one-factor model of Trivedi [24] with all items loading on one depression factor, the two-factor model of Bernstein [37] with a "depression" factor (all items) and a "somatic" factor (items 25, 26, and 28), the three-factor model of Rush [14] with a "mood/cognition" factor (items 5, 8, 10-14, and 17-22), an "anxiety/arousal" factor (items 6, 7, 23-28, and 30) and a "sleep" factor (items 1-4). Items 9, 15, 20, and 29 loaded on factors 1 and 2. Item 24 loaded on factors 2 and 3. The recently three-factor model of Wardenaar [33] with a "mood/cognition" factor (items 5-8, 10, 15-23, and 29), an "anxiety/somatic" factor (11-14, 25-28) and a "sleep" factor (items 1-4). Items 9, 24, and 30 loaded on factors 1 and 2. Finally, we also tested the fit of our exploratory factor solution.

In ordinal items with a non-normal distribution, as the ones in the inventory, it may be expected that the covariances underestimate the true amount of relations among variables. Therefore, we proceeded to estimate the models from the matrix of polychoric correlations [38]. The Mean and Variance corrected Weighted Least Squares (WLSMV) was applied to test the fit of the factor models.

Although a model with a non significant chi-square estimate is generally considered a good fitting model, Hu and Bentler recommended combinational rules to evaluate model fit [39]. Therefore, the following indices were analysed (values in parentheses denote goodness-of-fit standards): the Tucker-Lewis, non-normed fit index (TLI ≥ 0.95), the comparative fit index (CFI ≥ 0.95), and the root means square error of approximation (RMSEA ≤ 0.08). We also report the Akaike's Information Criterion (AIC), a relative fit index especially designed for comparing alternative factor models. The model with the smallest AIC value has the best fit. Used together, these indices provide a more conservative and reliable evaluation of the solution. The goodness of fit index (GFI), the adjusted goodness of fit index (AGFI), the relative fit index (RFI), and the normed fit index (NFI) were not used because these indices would likely be affected by the large sample size. Model comparisons were performed based on a practical improvement in model-fit approach (TLI difference ≥ 0.01) [40,41].

Internal consistency. Stratified Cronbach's coefficient [42] was used to assess the reliability in the presence of subscales. Subscale internal consistency estimations were conducted on the best-fit model observed following CFA.

Construct validity. Pearson product-moment correlations computed between the IDS-SR_30 _factors and the HDRS. We took into account the Cohen's criteria [43] to evaluate the substantive significance of correlations (large correlations are those > 0.50, medium correlations are from 0.30 to 0.49, and small correlations are from 0.10 to 0.29).

## Results

Sociodemographic and clinical characteristics of the total sample are presented in table 1. The final sample comprised 1595 patients, 553 men (34.6%) and 1042 women (65.3%) with a mean age of 47.7 years (Range 18-88). Most participants were married (61%), employed (45%), and living in urban residence (72%). The mean IDS-SR_30 _total score for the full sample was 34.85 (SD = 15) at first assessment and 15.34 (SD = 11) at second assessment. A paired T test indicated that the difference was statistically significant (t(1593) = 53.98, p < 0.001). Descriptive statistics were computed for all items as shown in Table 2. Each item was examined in terms of mean, standard deviation, skewness, and kurtosis. Univariate values approaching at least 2.0 for skewness and 7.0 for kurtosis indicate marked nonnormality [44]. Therefore, item 4 at first assessment and items 3, 4, 13/14, 18, and 27 at second assessment had questionable normality based on their skewness values, whereas Item 4 at first assessment and items 4 and 27 at second assessment had questionable normality based on their kurtosis values. With the exception of item 4, all items obtained a corrected item-total correlation that was higher than the rule of thumb minimum value of 0.20 [45].

**Table 1:** Sociodemographic and clinical characteristics of the study sample

Sociodemographic variables	n (%)
**Gender**	
Male	553 (34.6)
Female	1042 (65.3)
**Age**	47.73 (13.14)
≤ 30	155 (9.71)
31-50	780 (48.90)
> 50	660 (41.37)
**Employment Status**	
Employed	851 (45.0)
Student	45 (2.4)
Unemployed	189 (11.8)
Housework	383 (24.0)
Retired	254 (15.9)
**Civil Status**	
Single	315 (19.7)
Married	973 (61)
Widowed	111 (7)
Separated	196 (12.3)
**Education**	
Incomplete Primary Studies	296 (18.6)
Complete Primary Studies	529 (33.2)
Secondary Studies	506 (31.7)
University	264 (16.6)
**Lives**	
Living Alone	280 (17.6)
Living accompanied	1315 (82.4)
**Environment**	
Rural residence	446 (28)
Urban residence	1149 (72)

**Clinical variables**	**M (SD)**

Age First Episode	40.31 (13.15)
Duration Episode	14.2 (9.4)
Number Previous Episode IDS-SR_30 _at first assessment HDRS at first assessment	3.72 (2.9) 34.8 (15) 17.3 (8.3)

	**n (%)**

IDS-SR_30 _severity	
None (0-13)	200 (12.5)
Mild (14-25)	357 (22.4)
Moderate (26-38)	416 (26.1)
Severe (39-48)	347 (21.8)
Very severe (> 49)	275 (17.2)
HDRS severity	
None (0-13)	200 (12.5)
Mild (14-25)	286 (17.9)
Moderate (26-38)	352 (22.1)
Severe (39-48)	626 (39.2)
Very severe (> 49)	131 (8.2)

**Table 2:** Means (M), standard deviation (SD), skewness, kurtosis, and corrected item-total correlations (r_tot_) for all IDS-SR_30 _items

IDS-SR Items	First assessment				Second assessment			
		
	M (SD)	Skewness	Kurtosis	r_tot_	M (SD)	Skewness	Kurtosis	r_tot_
1. Initial insomnia	1.41 (0.99)	0.11	-1.03	0.50	0.59 (0.68)	0.93	0.54	0.43
2. Middle insomnia	1.44 (1.08)	0.13	-1.25	0.55	0.56 (0.77)	1.26	0.91	0.52
3. Early morning awakening	1.14 (1.15)	0.45	-1.28	0.56	0.33 (0.87)	2.19	4.46	0.47
4. Sleeping to much	0.22 (0.58)	2.99	9.27	0.03	0.20 (0.47)	2.56	7.55	0.16
5. Feeling sad	1.92 (0.91)	-0.33	-0.88	0.75	0.77 (0.70)	0.71	0.60	0.73
6. Feeling irritable	1.15 (0.80)	0.24	-0.49	0.49	0.54 (0.59)	0.66	0.10	0.58
7. Feeling anxious or tense	1.52 (0.78)	0.04	-0.40	0.57	0.72 (0.61)	0.48	0.54	0.63
8. Reactivity of mood	1.53 (0.93)	-0.01	-0.85	0.75	0.53 (0.65)	1.02	0.84	0.70
9. Diurnal variation of mood	1.04 (0.94)	0.52	-0.70	0.29	0.55 (0.69)	1.22	1.42	0.48
10. Quality of mood	1.62 (0.93)	-0.09	-0.87	0.63	0.65 (0.81)	1.14	0.66	0.68
11+12. Appetite disturbance	1.03 (0.87)	0.52	-0.40	0.57	0.36 (0.58)	1.63	2.85	0.49
13+14. Weight disturbance	1.07 (1.08)	0.57	-0.99	0.50	0.38 (0.74)	2.12	3.99	0.35
15. Concentration/decision-making	1.65 (0.87)	0.07	-0.81	0.72	0.80 (0.66)	0.47	0.25	0.68
16. Self criticism and blame	1.33 (0.89)	0.24	-0.64	0.67	0.53 (0.68)	1.23	1.47	0.68
17. Future pessimism	1.66 (0.83)	0.17	-0.77	0.72	0.83 (0.67)	0.58	0.66	0.68
18. Suicidal thoughts	0.77 (0.77)	0.77	0.15	0.62	0.21 (0.45)	2.15	5.11	0.55
19. Interest in people/activities	1.69 (0.97)	0.01	-1.12	0.76	0.68 (0.71)	0.96	1.12	0.73
20. Energy/fatigability	1.59 (0.85)	0.10	-0.67	0.75	0.77 (0.66)	0.63	0.72	0.71
21. Pleasure or enjoyment (not sex)	1.50 (0.82)	0.29	-0.52	0.74	0.67 (0.62)	0.62	0.29	0.72
22. Interest in sex	1.78 (1.03)	-0.25	-1.14	0.61	1.06 (0.92)	0.62	-0.38	0.52
23. Psychomotor retardation	1.13 (0.86)	0.41	-0.48	0.68	0.42 (0.58)	1.15	0.71	0.64
24. Psychomotor agitation	0.91 (0.78)	0.69	0.25	0.44	0.38 (0.58)	1.51	2.54	0.55
25. Aches and pains	1.05 (0.70)	0.37	0.18	0.47	0.60 (0.62)	0.69	0.37	0.57
26. Sympathetic arousal	1.00 (0.77)	0.41	-0.24	0.51	0.48 (0.60)	1.00	0.76	0.56
27. Panic/phobic symptoms	0.56 (0.83)	1.32	0.80	0.36	0.19 (0.47)	2.65	7.39	0.43
28. Constipation/diarrhoea	0.57 (0.78)	1.34	1.30	0.37	0.34 (0.59)	1.93	4.27	0.32
29. Interpersonal sensitivity	1.17 (0.95)	0.44	-0.70	0.54	0.54 (0.65)	1.08	1.14	0.58
30. Leaden paralysis/Physical energy	1.38 (0.87)	0.19	-0.62	0.65	0.66 (0.69)	0.88	0.67	0.65

Note: The SE of skewness was 0.06 at first and second assessment. The SE of kurtosis was 0.12 at first and second assessment.

### Principal component analysis (PCA)

The KMO measure produced a coefficient of 0.96, indicative of excellent sampling adequacy. Bartlett's test of Sphericity produced a figure of 21669.61 (P < 0.0001), indicating that the correlation matrix was unlikely to be an identity matrix and was therefore suitable for factor analysis. According to the Horn's parallel analysis three components were extracted. Factor loadings (after rotation) are displayed in Table 3. Tabachnick and Fidell [46] suggest that, in exploratory factor analysis, an item forms a part of a factor if its factor loading on that factor is at least 0.32 and at least 0.10 greater than its other factor loadings. Three items (items 3, 9, and 29) did not meet these criteria. Given that it is unlikely that the exclusion of the three items would yield a significant improvement in model fit in the subsequent CFAs, all further analyses were carried out on the IDS-SR_30_. The present three-factor solution (labelled Mood/Cognition, Anxiety/Somatic, and Sleep), which accounted for 49.87% of the variance, was more similar to the three-factor structure reported by Rush [14] than to the three-factor structure recently reported by Wardenaar [33]: there were divergences (different primary loading) in 5 items (items 3, 15, 23, 29, and 30) and 8 items (items 3, 6, 7, 11/12, 13/14, 24, 29, and 30), respectively.

**Table 3:** Results of a principal component analysis in the first assessment dataset (n = 1595)

IDS-SR Items	Component			Rush et al (1996)^a^	Wardenaar et al (2010)^b^
			
	1. Mood/cognition	2. Anxiety/somatic	3. Sleep		
1. Initial insomnia	0.47	0.46	**0.64**	3	3
2. Middle insomnia	0.53	0.48	**0.66**	3	3
3. Early morning awakening	**0.58**	0.39	**0.55**	3	3
4. Sleeping to much	0.06	0.12	**-0.64**	3	3
5. Feeling sad	**0.81**	0.46	0.38	1	1
6. Feeling irritable	0.42	**0.63**	0.30	2	1
7. Feeling anxious or tense	0.49	**0.71**	0.39	2	1
8. Reactivity of mood	**0.81**	0.44	0.33	1	1
9. Diurnal variation of mood	0.30	0.24	0.16	-	-
10. Quality of mood	**0.71**	0.36	0.32	1	1
11+12. Appetite disturbance	**0.59**	0.46	0.07	1	2
13+14. Weight disturbance	**0.53**	0.40	0.07	1	2
15. Concentration/decision-making	**0.78**	0.47	0.29	2	1
16. Self criticism and blame	**0.72**	0.48	0.22	1	1
17. Future pessimism	**0.78**	0.49	0.29	1	1
18. Suicidal thoughts	**0.64**	0.50	0.21	1	1
19. Interest in people/activities	**0.84**	0.46	0.31	1	1
20. Energy/fatigability	**0.81**	0.49	0.26	1	1
21. Pleasure or enjoyment (not sex)	**0.83**	0.41	0.27	1	1
22. Interest in sex	**0.69**	0.36	0.28	1	1
23. Psychomotor retardation	**0.74**	0.43	0.23	2	1
24. Psychomotor agitation	0.33	**0.69**	0.32	2	1/2
25. Aches and pains	0.44	**0.59**	0.07	2	2
26. Sympathetic arousal	0.43	**0.73**	0.22	2	2
27. Panic/phobic symptoms	0.28	**0.63**	0.04	2	2
28. Constipation/diarrhoea	0.33	**0.52**	0.00	2	2
29. Interpersonal sensitivity	**0.55**	**0.52**	0.09	2	1
30. Leaden paralysis/Physical energy	**0.69**	0.54	0.13	2	1/2
**Eigenvalue (in real data)**	10.89	1.75	1.33		
**Eigenvalue (randomly generated)**	1.25	1.22	1.19		

Factor loadings and eigenvalues.

Note: IDS-SR = Inventory of Depressive Symptomatology Self Report. Boldface indicates the primary loading for each item.

^a ^Components on which each item had its highest loading in PCA results by Rush et al (1996). An underlined number indicates that the item loads on a different component in this study.

^b ^Components on which each item had its highest loading in PCA results by Wardenaar et al (2010). An underlined number indicates that the item loads on a different component in this study.

### Confirmatory factor analyses (CFAs)

Fit statistics for the factor models are shown in Table 4. Although all models showed RMSEA values that are considered acceptable (< 0.08), the one-factor and two-factor solutions failed to provide good fit taking the other indices into account. Considering the findings collectively, all three-factor models seem adequate for the current sample. However, as the Hu and Bentler's guidelines for retaining a hypothesized model recommended, the factor structure posited by Wardenaar [33] received more support than the other three-factor structures because obtained the lowest AIC value and its CFI and TLI values reached the rule of thumb minimum value of 0.95. Furthermore, the TLI value was 0.01 greater (0.95 vs. 0.94). The correlations between the Wardenaar's factors were all positive and statistically significant (p < 0.01): Mood/Cognition * Anxiety/Somatic = 0.76; Mood/Cognition * Sleep = 0.78; and Anxiety/Somatic * Sleep = 0.71. We decided to retain Wardenaar's model for further analyses.

**Table 4:** CFA of five factor models of the IDS-SR_30 _in the follow-up dataset (n = 1594)

Model	Source	*Df*	χ^2 ^	TLI	CFI	RMSEA (90% CI)	AIC
1-factor	e.a. Trivedi et al. (2004)	350	3793.5	0.93	0.94	0.079 (0.076-0.081)	70462.8
2-factor	Bernstein et al. (2006)	347	3692.2	0.93	0.94	0.078 (0.076-0.080)	70339.9
3-factor	Rush et al. (1996)	342	3214.3	0.94	0.95	0.073 (0.070-0.075)	70018.2
**3-factor**	**Wardenaar et al. (2010)**	**344**	**2774.2**	**0.95**	**0.96**	**0.067 (0.064-0.069)**	**69926.9**
3-factor	PCA, present study	344	3245.4	0.94	0.95	0.073 (0.070-0.075)	69928.3

Note: IDS-SR_30 _= Inventory of Depressive Symptomatology Self Report; Df = degrees of freedom; CFI = Comparative Fit Index; TLI = Tucker-Lewis Index; RMSEA = Root Mean Square Error of Approximation; 90% CI = 90% confidence interval of the RMSEA. AIC = Akaike Information Criteria.

Indices that indicate the best model fit are printed in bold font.

### Internal consistency

Prior to examining the internal consistency and convergent validity of the IDS-SR_30_, the three items that had cross-loadings (9, 24, and 30) were assigned to the subscale with which they had the highest factor loading on the CFA to compute the subscale scores. Thus, items 9 and 30 were assigned to the Mood/Cognition sub-scale, whereas item 24 was assigned to the Anxiety/Somatic sub-scale.

The results of the reliability analysis are shown in table 5. The stratified overall Cronbach's coefficient was 0.94 at first and second assessment. Cronbach's α coefficient ranged from 0.54 to 0.93 in the three subscales derived from the Wardenaar's factor model. If item 4 was deleted, the Cronbach's α of the Sleep sub-scale would be 0.73 and 0.64 at first assessment and second assessment, respectively.

**Table 5:** Cronbach's α coefficient of the IDS-SR_30 _(total and sub-scale scores)

**α**	**Mood/Cognition**	**Anxiety/Somatic**	**Sleep**	**IDS-SR_30_**
	
First assessment	0.93	0.77	0.57	0.94*
Second assessment	0.93	0.75	0.54	0.94*

Note: *Stratified-alpha Coefficient

### Convergent validity

The IDS-SR_30 _would show adequate convergent validity if the total and subscale scores correlated significantly with the HDRS. As can be seen in Table 6, the correlations between Mood/Cognition, Anxiety/Somatic, Sleep, the IDS-SR_30 _total score and the HDRS were all significant and large at both assessments.

**Table 6:** Correlations between the IDS-SR_30 _(total and sub-scale scores) and the HDRS at first and second assessment

**HDRS**	**Mood/Cognition**	**Anxiety/Somatic**	**Sleep**	**IDS-SR_30_**
	
First assessment	0.82*	0.70*	0.70*	0.85*
Second assessment	0.82*	0.67*	0.64*	0.85*

Note: *Correlation significant at *p *< 0.001

## Discussion

The main conclusion of our study is that the Spanish version of the IDS-SR has good psychometric properties and it is a useful tool for evaluating depressive symptomatology in Spanish population.

The rationale for and psychometric properties of the IDS (Clinician and Self-rated version) have been previously discussed [14,16]. Both versions of the scale attempt to address limitations of the HDRS and MADRS which do not cover all of the diagnostic symptoms criteria for Major Depressive Disorder (MDD) or depressive subtypes (e.g. melancholic or atypical features).

Good evidence of the correspondence between individual items and the total IDS-SR_30 _score was found. All items are consistent with the scale except for item that measures hypersomnia that showed no correlation with the total score. Internal consistency of the Spanish-language version of the IDS-SR_30 _was good (α = 0.94). Other adaptations and validity studies confirmed the high consistency of this instrument: (α = 0.77) [14], (α = 0.79) [22], (0.83) [46], (α = 0.92) [24], (α = 0.93) [25]. In our study, the correlation between the IDS-SR (total and sub-scale scores) and the HDRS at both assessments were strong (0.85). These results were similar compared to previous findings [14,16,47]. This indicates a good convergent validity. The present study used CFA to identify the best-fitting structural model of the IDS-SR. The unidimensional structure was not supported by exploratory or confirmatory analisys. Although an initial PCA indicted that our 3-factor solution was more similar to the 3-factor structure reported by Rush [14] the CFA identifies the Wardenaar factor structure [33] as the best fitting model of the IDS-SR_30_.

Our results have some implications. On the one hand IDS-SR_30 _do not seem to be a unidimensional measure of depression severity and, on the other hand those items that measures atypical features (hypersomnia, appetite and weight increase, interperpersonal sensitivity and leaden paralysis) and some of the items that feature endogenous/melancholic depression (diurnal mood variation, appetite and weight decrease) seems to be the more psychometrically problematic items. More analysis to find out a different and more complete factorial structure that includes depressive subtypes is needed. The heterogeneity of patients suffering from depressive disorders adds complexity to the study and design of good and well suited instruments for measure depressive symptomatology.

A strength of the present study is the large and representative sample which makes the results generalizable to depressive Spanish outpatients. Second, all the analyses were conducted in patients that met MDD criteria and 6-8 weeks of antidepressant treatment and allows us to have all the severity degrees represented. Third, a 3-factor model was displayed in the statistical analysis, as recommended by the authors of the original version [14]. This fact allows the comparison of the results from similar studies. The main limitation of this study is that we did not administered the questionnaire in primary care. Further analysis in several settings should reveal whether our results could be generalized to depressive inpatients or primary care settings.

## Conclusions

In conclusion, our findings from this first validation study indicate that the Spanish version of the IDS-SR have highly acceptable psychometric properties, corroborating results of the original English version [14,48] and other studies and translations. The IDS-SR_30 _is a simple and easy-to-use tool, suitable for clinical and research use with satisfactory properties in the Spanish population.

## Conflict of interests

The RESIST project was possible due to an unrestricted grant from Almirall Spain. The sponsor had no role in the design and conduct of the study, analysis, writing of the report or in the decision to submit the paper for publication.

MR has received grant/support research from Almirall. SA is employee of Almirall Medical Department. The rest of the authors report no competing interest.

## Authors' contributions

MG, MR and SA designed and wrote the protocol; JVL, JA and NB performed the statistical analysis. MG, JVL, NB and MR wrote the draft of the manuscript. All authors contributed to, revised and approved the final manuscript.

## Pre-publication history

The pre-publication history for this paper can be accessed here:

http://www.biomedcentral.com/1471-2288/11/131/prepub
